# Dissociation between changes in sprinting performance and Nordic hamstring strength in professional male football players

**DOI:** 10.1371/journal.pone.0213375

**Published:** 2019-03-14

**Authors:** Luis Suarez-Arrones, Pilar Lara-Lopez, Pablo Rodriguez-Sanchez, Jose Luis Lazaro-Ramirez, Valter Di Salvo, Marc Guitart, Cristobal Fuentes-Nieto, Gil Rodas, Alberto Mendez-Villanueva

**Affiliations:** 1 Department of Sport and Informatics, Section of Physical Education and Sport, Pablo de Olavide University, Sevilla, Spain; 2 Football Performance & Science Department, ASPIRE Academy, Doha, Qatar; 3 Department of Movement, Human and Health Sciences, University of Rome “Foro Italico”, Rome, Italy; 4 Medical and Performance Department, Futbol Club Barcelona, Barcelona, Spain; 5 Cordoba Club de Futbol, SAD, Cordoba, Spain; Universidad Europea de Madrid, SPAIN

## Abstract

The aim of the present study was to evaluate the consequence of implementing a Nordic Hamstring exercise (NHE) protocol during the first 15 to 17 weeks of the season to assess the effect on sprinting and NHE strength (NHEs) in professional football players. The study examined 50 healthy male professional football players (age 18.8±0.8yr; height 176.8±6.9cm; weight 71.3±5.7kg) belonging to 3 of the reserve squads of three Spanish La-Liga clubs divided in 2 intervention teams [Nordic-Group1 (NG-1) and Nordic-Group2 (NG-2, extensive experience in NHE)] and 1 team as a control-group (CG). NHEs and linear sprint (T5, T10, T20-m) were evaluated at the beginning of the season and at the end of an intervention period of conditioning and football training, supplemented with a NHE protocol (24 sessions for NG-1 and 22 sessions for NG-2) or without using the NHE at all (CG). Sprint times were substantially improved in all groups (ES from -2.24±0.75 to -0.60±0.37). NHEs was enhanced absolute and relative to body-mass only in NG-1 after the training period (ES from 0.84±0.32 to 0.74±0.26), while in the NG-2 there were only improvements in average NHEs relative to body-mass (ES = 0.39±0.36). The improvements in T20-m were substantially greater in NG-2 *vs*. NG-1, and there were no differences in sprint performance changes between NG-1 and CG. Changes in sprinting performance and NHEs were unrelated. NHEs was largely correlated with the body-mass of the players. Results indicate that the improvements in sprint are not dependent on the NHEs changes, with no relationships between NHEs and sprint performance, and between sprint changes and changes in NHEs.

## Introduction

Hamstring muscle tears are the most common injury subtype in male football players, and are associated with significant time loss and high financial costs for the player and clubs [1, 2]. Recent research findings on the links between eccentric strength, hamstring muscles fascicle length and injury risk have promoted the use of Nordic Hamstring exercise (NHE) to enhance maximal eccentric hamstring strength (EHS), measured during the NHE, and increase the biceps femoris long head fascicle length as an effective strategy to reduce the risk of a future hamstring strain injury [3, 4]. However, and despite its simplicity and proposed efficacy, it appears that the vast majority of professional clubs do not include the NHE protocol as part of their injury prevention strategy [5–7]. This might be due to sports medicine and coaching professionals labeling the NHE as ineffective for injury prevention and performance [8]. In regards to performance, two recent studies investigated changes in sprint performance after an injury-prevention NHE protocol, showing significant improvements in sprint performance [7, 9] and EHS using the NHE [10]. Ishoi et al. (2017) reported that 10-weeks of training with NHE resulted in significant improvements in 10 m sprint performance (2.6%) and repeated-sprint performance (1.8%) in amateur male football players. On the other hand, Krommes et al. (2017) found significant improvements in 5-m (9.4%) and 10-m (5.8%) sprint performance in elite male Danish football players after the 10-week NHE protocol. Considering that straight sprinting is one of the most game-deciding physical action in professional football [11] and the predominant injury condition in hamstring strain injuries [9, 12] the inclusion of the NHE protocol can be a very time efficient and effective training intervention targeting both the injury prevention and performance sites. However, the limited information currently available, only 2 short reports, about the effects of NHE on sprinting performance makes it difficult to give solid, evidence-based recommendations. In addition, previous experience with the NHE could influence the results as well as the effect of body mass (BM) on NHE strength (NHEs) that has important implications when monitoring and comparing players over long periods [13], and we must be mindful that there does come a time when it will be very difficult for the players to improve the NHEs due to the effect of the BM in the force applied. On that basis, the aim of the present study was to evaluate the impact of implementing a NHE protocol during the first weeks of the season on sprinting and NHE performance in professional football players from three different squads.

## Materials and methods

### Subjects

This study examined 50 healthy male professional football players (age 18.8 ± 0.8 yr; height 176.8 ± 6.9 cm; weight 71.3 ± 5.7 kg) belonging to 3 of the reserve squads of two of Spanish La Liga Santander clubs and one of La Liga 123 club. All the players trained ~ 8 hours of football training plus 1 or 2 competitive games per week. To be eligible for the study, players were required to meet the following criteria: (i) to have a current professional contract with the one of the reserve squads of the club; and (ii) to be injury free at the moment of the study. The purpose and experimental protocol was explained to the players and written informed consent was obtained from the players (or tutor for players under 18). The present study was approved by the local Institutional Research Ethics Committee (Qatar Antidoping Lab), and conformed to the recommendations of the Declaration of Helsinki.

### Experimental procedures

The present study used a controlled repeated-measures research design to investigate the changes in EHS using the NHE and the linear sprint in response to an intervention of football training, supplemented with a NHE protocol 1 or 2 times a week in male professional football players. EHS and linear sprint were evaluated at the beginning of the season and at the end of the intervention period. Teams and players were divided in 2 intervention teams [Nordic Group 1 (NG-1) and Nordic Group 2 (NG-2)] and 1 team as a control group (CG).

Total training volume (training sessions, friendly games and official games) for NG-1 during the intervention period (17 weeks) was 6911.5 min. Approximately 1.8% of that training time was devoted to the NHE (24 sessions in total). Players from NG-1 had some previous experience with the NHE (i.e., players were randomly exposed to training sessions including the NHE the season prior to the study). However, none of the players were previously subjected to a systematic (i.e., weekly) exposure to the NHE. Total training volume (training sessions, friendly games and official games) for NG-2 during the intervention period (15 weeks) was 6296.1 min, with approximately ⁓ 1.6% of this time employed training NHE (22 sessions of the NHE). Players from NG-2 had large previous experience with the NHE in the last years, training with this exercise at least once a week during the previous season. Control group completed regular conditioning and football training according with the original plan, which includes neuromuscular training, conditioning and football training (with friendly and official games) during 15 weeks. Control group never trained with NHE during the previous season and most of the players have no previous experience with the exercise. During the intervention time, the control group was not allowed to use the NHE at all. Total training volume (training sessions, friendly games and official games) for CG during the intervention period (15 weeks) was 5897.3 min. All players were familiar with the procedures of both tests and were asked not to perform any strenuous exercise ~ 48 h before the testing sessions. The performance tests were carried out at the beginning of the training session and after a standardized warm-up. All of tests were performed at the same time of the day and in the same order: first the sprint test and then the NHE.

### The Nordic hamstring protocol

The Nordic hamstring protocol consisted of 24 (NG-1) and 22 sessions (NG-2) of the NHE performed during the initial part of the session on the field, or included as a part of the neuromuscular training session in the gym before the training on the field. Table 1 shows the progressive eccentric strengthening program employing the NHE during the intervention period.

**Table 1:** Progressive eccentric strengthening program using the Nordic hamstring exercise.

Week	Sessions per week	Sets and repetitions
1	1	2 x 5
2	2	2 x 6
3	2	3 x 6
4	2	3 x 8
5–8	2	3 x 10
9—Last	1	3 x 10

### Eccentric knee flexor strength

The assessment of eccentric hamstring strength using the Nordic hamstring exercise was carried out using a similar field-testing device and associated software (Acceleration Leg Curl/Extension, NeuroExcellence, Porto, Portugal) to what has been reported previously [14]. Participants were positioned in a kneeling position over a padded board, with the ankles secured superior to the lateral malleolus by individual ankle braces, which were secured atop custom made uniaxial load cells (Sensocar, Barcelona, Spain). The ankle braces and load cells were secured to a pivot, which allowed the force to always be measured through the long axis of the load cells. Following a warm up set, participants were asked to perform one set of three maximal bilateral repetitions of the Nordic hamstring exercise. Participants were instructed to gradually lean forward at the slowest possible speed while maximally resisting this movement with both lower limbs while keeping the trunk and hips in a neutral position throughout, and the hands held across the chest. Following each attempt a visual analogue scale was given to assess the level of pain that was experienced. None of the participants reported any pain during testing. Verbal encouragement was given throughout the range of motion to ensure maximal effort. The peak force for each of the three repetitions was averaged for all statistical comparisons (_AVG_EHS). The highest peak force recorded in any of the three repetitions was also retained for further analysis (_PEAK_EHS).

### Sprinting performance

Running speed was evaluated by a 20-m sprint time (standing start) with 5-m and 10-m split times. The 20-m sprint test was conducted outdoors with suitable weather conditions (i.e., sunny and negligible wind) on an artificial turf field. The front foot was placed 0.5 m before the first timing gate, and players started when ready eliminating reaction time. Time was recorded with photoelectric cells (Witty, Microgate, Bolzano, Italy). The 20-m sprint was performed two times, separated by at least 1 min of passive recovery. The best time was considered for subsequent analysis.

### Statistical analyses

Data are presented as means ± standard deviation (SD). The normality of distribution of the variables in the Pre-test and the homogeneity of variance across groups were verified using the *Shapiro-Wilk* test and *Levene*’s test, respectively. Data were analyzed using a 2 x 2 factorial ANOVA using one between factor and one within factor (Pre *vs*. Post). In addition, all data were first log-transformed to reduce bias arising from non-uniformity error. Possible differences or changes in sprint performance and NHEs values within- and between-groups were analysed for practical significance using magnitude-based inferences by pre-specifying 0.2 between-subject SDs as the smallest worthwhile effect [15]. The standardized difference or effect size (ES, 90% confidence limit [90%CL]) in the selected variables was calculated. Threshold values for assessing magnitudes of the ES (changes as a fraction or multiple of baseline standard deviation) were <0.20, 0.20, 0.60, 1.2 and 2.0 for trivial, small, moderate, large and very large respectively [15]. Quantitative chances of higher or lower changes were evaluated qualitatively as follows: <1%, almost certainly not; 1 −5%, very unlikely; 5−25%, unlikely; 25−75%, possible; 75−95%, likely; 95−99%, very likely; >99%, almost certain [15]. A substantial effect was set at >75% [16]. If the chance of both higher and lower values was > 5%, the true difference was assessed as unclear [15]. Otherwise, we interpreted that change as the observed chance. For further analysis, players were divided into a group of players with the greatest improvements in sprint performance, based on a small standardized ES (i.e. 0.2 x SD) from the sprint changes average. Pearson's product-moment correlation analysis was also used to investigate the association between all variables. The following criteria were adopted to interpret the magnitude of the correlation (r) between the different measures: ≤ 0.1, trivial; 0.1–0.3, small; > 0.3–0.5, moderate; > 0.5–0.7, large; > 0.7–0.9, very large; and > 0.9–1.0, almost perfect. If the 90% confidence limits overlapped positive and negative values, the magnitude were deemed unclear; otherwise that magnitude was deemed to be the observed magnitude.

## Results

### Changes following the training process

**Fig 1:**
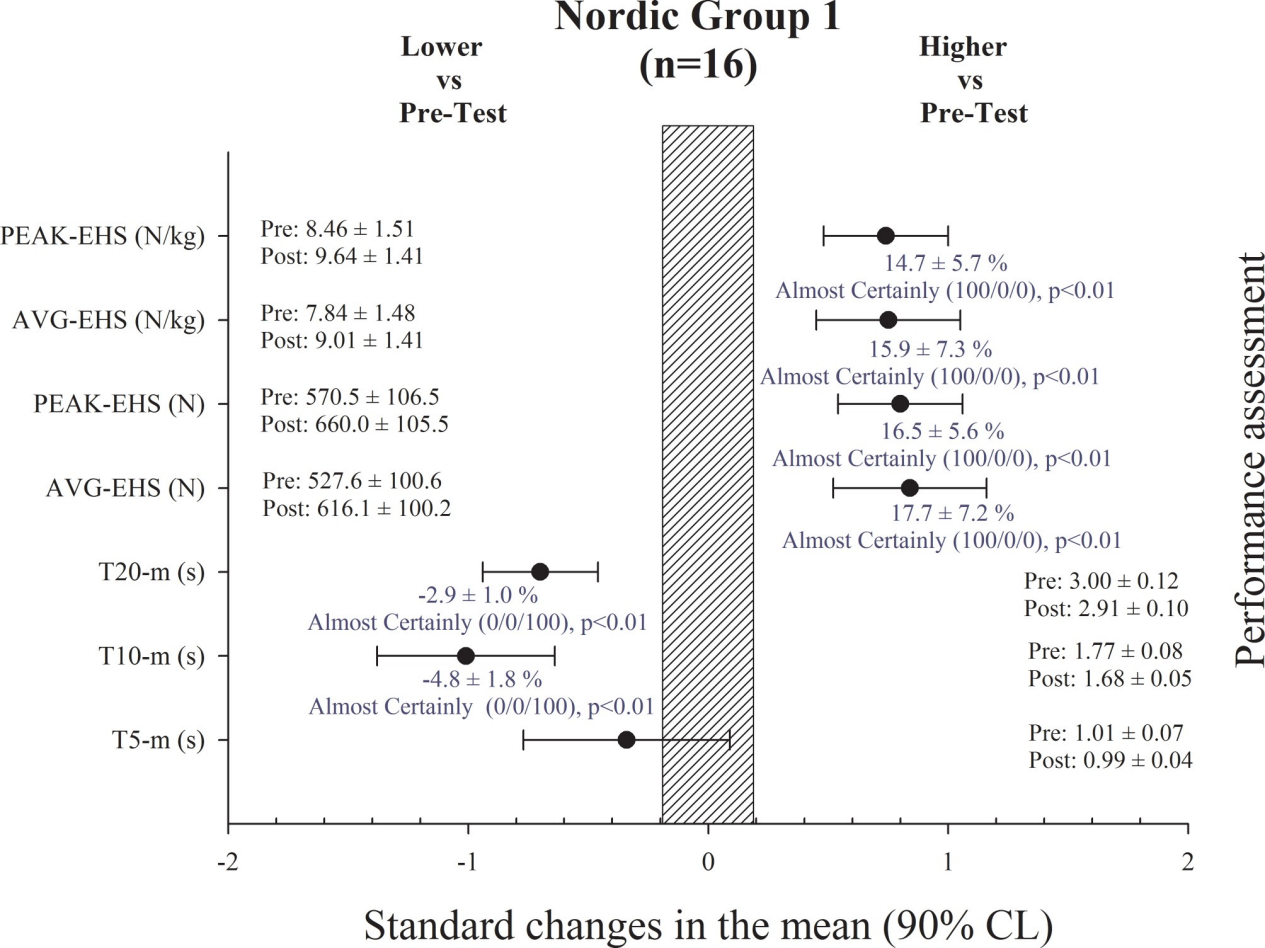
Performance assessment results in the Nordic Hamstring group 1. T: time; AVG-EHS: eccentric hamstring strength average; PEAK-EHS: eccentric hamstring strength peak.

**Fig 2:**
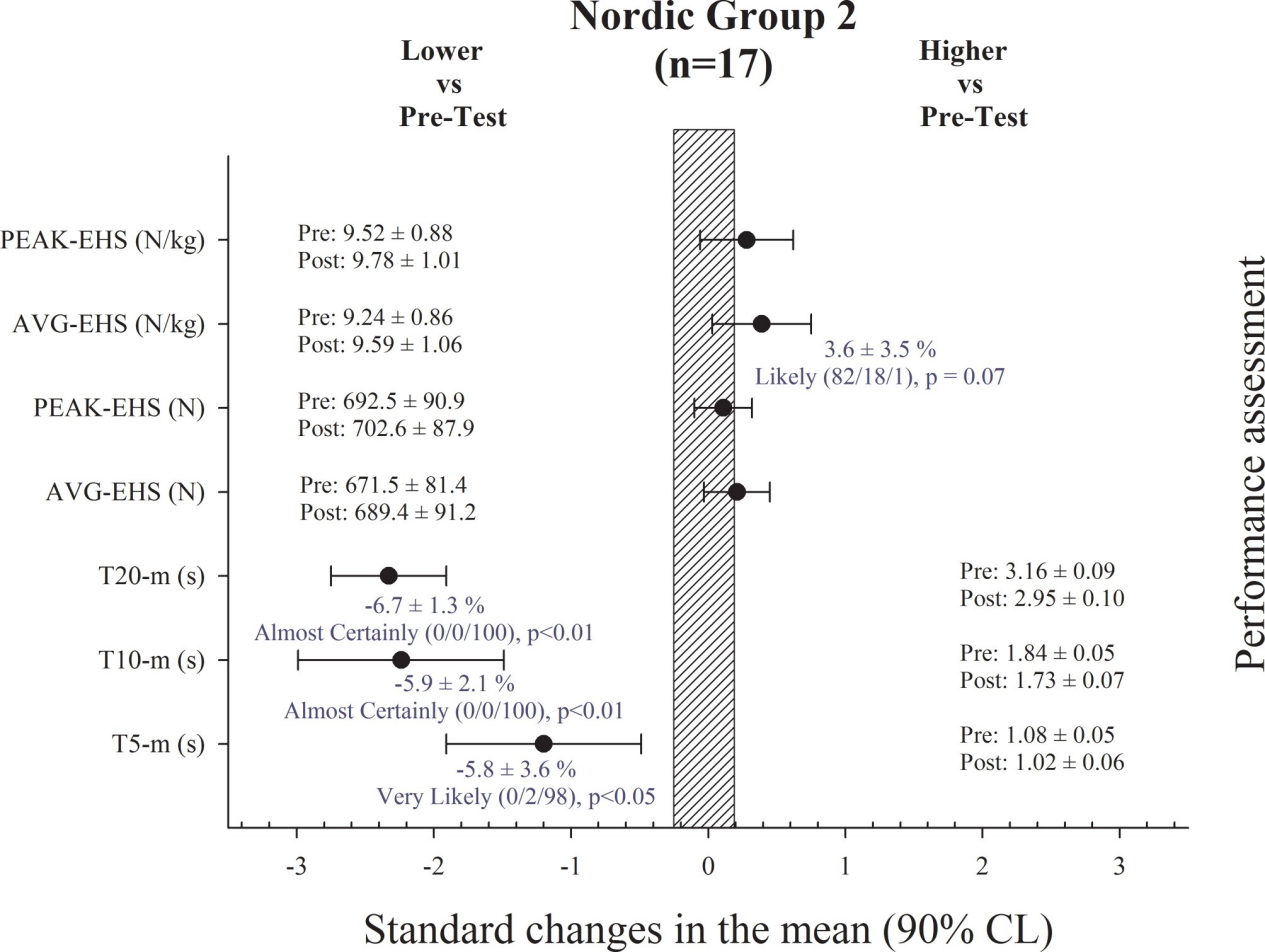
Performance assessment results in the Nordic Hamstring group 2. T: time; AVG-EHS: eccentric hamstring strength average; PEAK-EHS: eccentric hamstring strength peak.

**Fig 3:**
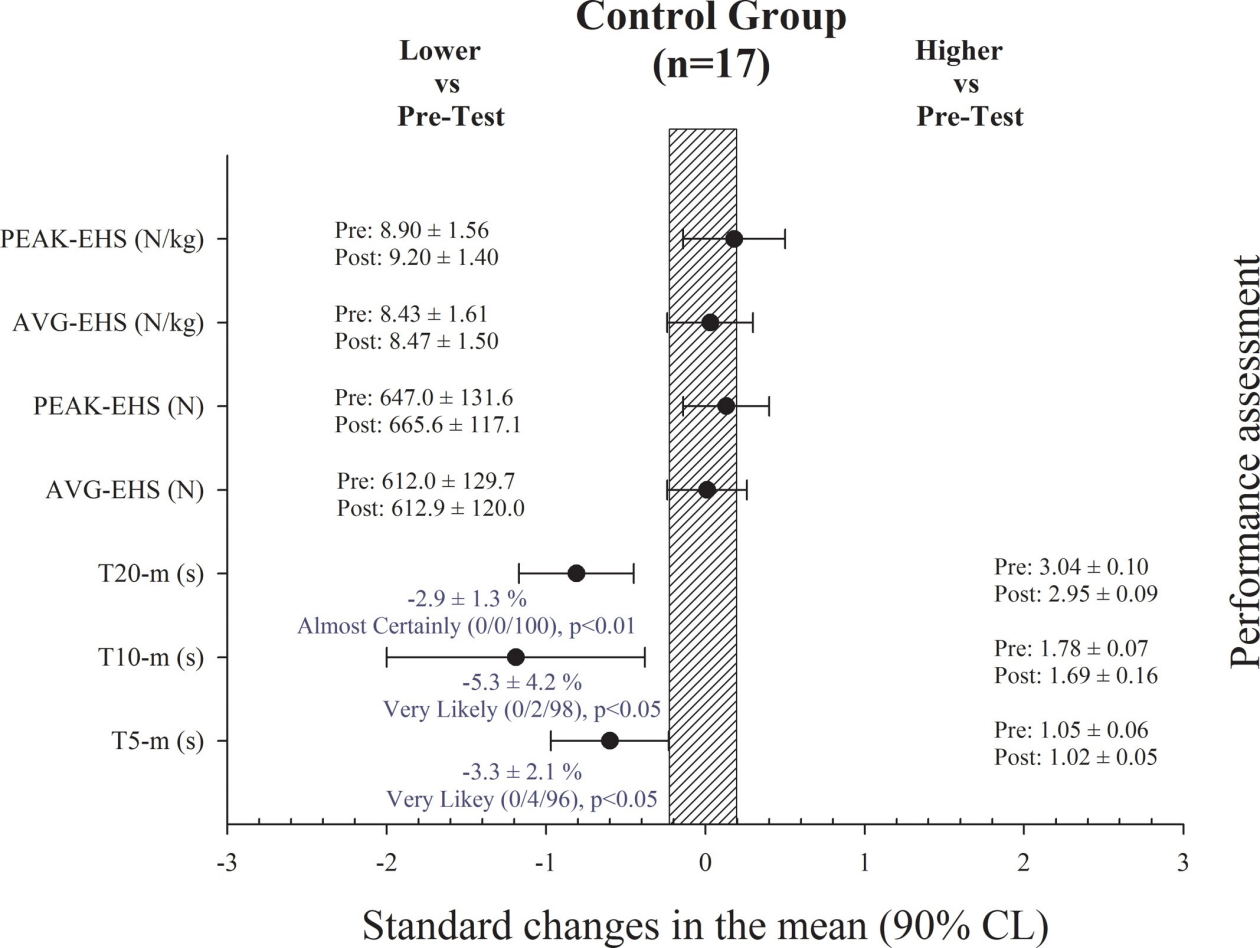
Performance assessment results in the control group. T: time; AVG-EHS: eccentric hamstring strength average; PEAK-EHS: eccentric hamstring strength peak.

Performance test results in NG-1, NG-2 and CG are shown in Figs 1, 2 and 3, respectively. There were no changes in BM of the players in any of the groups after the training period.

Sprint times were substantially improved in the NG-1 after the training period in 10 and 20-m split times (ES = -1.01 ± 0.37 and ES = -0.70 ± 0.24, respectively). EHS in the NHE was substantially increased after the training period in _AVG_EHS absolute and relative to BM (ES = 0.84 ± 0.32 and ES = 0.75 ± 0.30, respectively), and in _PEAK_EHS absolute and relative to BM (ES = 0.80 ± 0.26 and ES = 0.74 ± 0.26, respectively) (Fig 1). Sprint times also substantially improved in the NG-2 after the training period in 5, 10 and 20-m split times (ES = -1.20 ± 0.71, ES = -2.24 ± 0.75 and ES = -2.33 ± 0.42, respectively). EHS in the NHE was substantially increased after the training period only in _AVG_EHS relative to BM (ES = 0.39 ± 0.36) (Fig 2), with no changes in absolute values. Lastly, sprint times were improved in the CG after the training period in 5, 10 and 20-m split times (ES = -0.60 ± 0.37, ES = -1.19 ± 0.81 and ES = -0.81 ± 0.36, respectively), while there were no changes in EHS (Fig 3).

Results from the between-group analysis in sprint performance are illustrated in Table 2. The improvements in T20-m were substantially greater in NG-2 in comparison with NG-1, while there were no substantial differences between groups in T10-m. There were no differences in sprint performance changes between NG-1 and CG. The improvements in T20-m were substantially greater in NG-2 in comparison with CG, while there were no substantial differences between groups in T5 and T10-m.

**Table 2:** Results from the between-group analysis in sprint performance. Standard changes in mean (90%CL).

	Between group mean difference of changes in sprint performance		
	Nordic Group I *vs*. Nordic Group II	Control Group *vs*. Nordic Group I	Control Group *vs*. Nordic Group II
T5-m	-0.55 ± 0.70 (Likely; p = 0.19)	0.18 ± 0.55	-0.46 ± 0.79
T10-m	-0.27 ± 0.63	0.01 ± 0.86	-0.29 ± 1.02
T20-m	-0.90 ± 0.36 (Almost Certainly; p<0.01)	-0.01 ± 0.42	-1.06 ± 0.46 (Almost Certainly, p<0.01)

### Relationships between the different parameters evaluated

When data from three groups were pooled, both _AVG_EHS as well as _PEAK_EHS in the NHE was largely correlated with BM (r = 0.57 (0.34 to 0.74) and r = 0.62 (0.40 to 0.77), respectively). There were unclear correlations between NHEs (absolute and relative to BM) and sprint performance.

### Individual relationships between sprint changes and eccentric hamstring strength

Correlations between changes in sprint performance and NHEs are shown in Table 3. Unclear correlations were found in the NG-1 and NG-2 analysed in isolation, when the NG-1 and NG-2 players were pooled together and when only the players with the greatest improvements in sprint performance were analysed.

**Table 3:** Relationships between sprint improvements and enhancements in NHEs (all p>0.05).

		**Nordic Group 1 (n = 16)**		
*Variable*	_AVG_EHS (N)	_PEAK_EHS (N)	_AVG_EHS (N/kg)	_PEAK_EHS (N/kg)
T5-m	-0.14 (-0.54; 0.30)	-0.16 (-0.55; 0.29)	-0.12 (-0.52; 0.33)	0.14 (-0.31; 0.53)
T10-m	-0.13 (-0.54; 0.31)	-0.13 (-0.53; 0.32)	-0.18 (-0.56; 0.27)	-0.13 (-0.53; 0.32)
T20-m	0.13 (-0.32; 0.53)	0.14 (-0.31; 0.53)	0.13 (-0.32; 0.52)	-0.16 (-0.55; 0.29)
		**Nordic Group 2 (n = 17)**		
*Variable*	_AVG_EHS (N)	_PEAK_EHS (N)	_AVG_EHS (N/kg)	_PEAK_EHS (N/kg)
T5-m	-	-	-0.30 (-0.67; 0.20)	-
T10-m	-	-	-0.43 (-0.75; 0.06)	-
T20-m	-	-	0.13 (-0.31; 0.54)	-
		**Nordic Group (all players, n = 33)**		
*Variable*	_AVG_EHS (N)	_PEAK_EHS (N)	_AVG_EHS (N/kg)	_PEAK_EHS (N/kg)
T5-m	-0.17 (-0.44; 0.13)	-0.12 (-0.40; 0.18)	-0.20 (-0.46; 0.10)	0.14 (-0.42; 0.15)
T10-m	-0.22 (-0.48; 0.08)	-0.18 (-0.45; 0.12)	-0.28 (-0.53; 0.01)	-0.25 (-0.50; 0.05)
T20-m	0.20 (-0.10; 0.46)	0.26 (-0.04; 0.51)	0.12 (-0.18; 0.40)	0.17 (-0.13; 0.44)
				
		**Players with the greatest improvements in sprint (n = 13)**		
*Variable*	_AVG_EHS (N)	_PEAK_EHS (N)	_AVG_EHS (N/kg)	_PEAK_EHS (N/kg)
T5-m	-0.38 (-0.73; 0.12)	-0.31 (-0.68; 0.20)	-0.42 (-0.75; 0.08)	-0.33 (-0.70; 0.18)
T10-m	-0.35 (-0.71; 0.15)	-0.33 (-0.70; 0.18)	-0.38 (-0.73; 0.12)	-0.35 (-0.71; 0.15)
T20-m	-0.01 (-0.48; 0.47)	-0.02 (-0.50; 0.46)	-0.12 (-0.57; 0.38)	-0.14 (-0.58; 0.36)
				

NHEs: Nordic hamstring exercise strength; EHS: eccentric hamstring strength; AVG: average; N: Newton.

## Discussion

The present study analysed the effects of combined football and NHE training during the first weeks of the season on sprinting and EHS changes with professional football players. The main findings of the present study were: i) sprint times were improved in all groups; ii) EHS in the NHE was enhanced only in NG-1, while in the NG-2 there were only improvements in _AVG_EHS relative to BM; iii) both _AVG_EHS as well as _PEAK_EHS in the NHE was largely correlated with BM; iv) there were unclear correlations between EHS and sprint performance, and between sprint changes and changes in EHS.

Sprint acceleration is a key component of performance in football [11] and constitutes the primary hamstring injury mechanism [2, 17]. The results of the present study showed improvements in sprint performance after the NHE protocol, in line with previous findings using a similar protocol [7, 9]. However, our data also showed sprint times substantially reduced in CG, which did not perform NHE at all. Sprint performance in football players can be improved after hamstring muscle strength training in isolation or in concurrence with other training methods [7, 9, 18, 19], without employing any specific strength exercise for the hamstring muscle group in adult [20, 21] and young football players [22, 23], or by including no strength exercises but sprints [24]. In addition, present findings indicate that a combined football training program (including specific conditioning and neuromuscular training) without a systematic emphasis on either eccentric hamstring strength and/or sprinting can induce substantial changes in sprinting performance (CG, Table 3). In this regard, football players perform a high number of accelerations [25], jumps, turns and change of direction actions [26] with high neuromuscular load during football trainings and matches. Thus, it could be argued that the daily repetition of these actions during the regular football sessions can likely maximize player’s acceleration potential [27], and this could be another reason why football players can improve sprint performance over 0–20 m without having implemented training sessions with the NHE.

Previous studies reported gains in EHS after NHE training in football players following different intervention protocols (1–3 times [9, 28] *vs*. 2 times per week [29]). These improvements in EHS may suggest that the potential mechanism for improved sprint performance it could be as a result of an increased horizontal ground reaction force production [9]. Our data revealed that there were not clear relationships between NHEs and sprint performance, neither were relationships between changes in NHEs and changes in sprint performance. In addition, the present results showed that despite of being trained with a NHE protocol the NG-2 did change the _AVG_EHS and _PEAK_EHS (only *likely* changes in _AVG_EHS relative to BM). This lack of improvement in EHS was concurrent with substantial improvements, which were greater than the NG-1, in sprint performance (T5 and T20-m, Table 2). Based on the above, a program based only in an isolated knee flexor eccentric strengthening exercise may have positive effects in terms of hamstring injury prevention in amateur football players [30], but NHEs does not seem to be an essential element to improve sprint performance in trained professional football players.

The effect of BM on NHEs has important implications when monitoring and comparing players over long periods, due changes in BM may occur [13]. Our results showed that players from NG-2 substantially increased their _AVG_EHS relative to BM after the training period, but their absolute NHEs remained the same. In line with a previous study our results suggest a likely effect of BM on NHEs because when leaning forward during the NHE, players’ BM probably affect the force applied to the dynamometers showing values that are likely not the true EHS [13]. Furthermore, players from NG-2 had extensive experience with the NHE, training with this exercise at least once a week throughout the entire last season. Therefore, we must be mindful that there does come a time when it will be very difficult from the players to improve the NHEs, mainly due to the great influence of the BM in the force applied. Together with the previous experience with the NHE, this can explain why this group of professional players did not improve their absolute values of NHEs after 22 sessions training with the exercises. On that basis, and in order to continue increasing the EHS in the football players, it appears necessary to employ different stimulus. While Nordic hamstring exercise can be recommended when the goal is to target semitendinosus (ST) and biceps femoris short head (BFs), other knee-dominant exercises such as the flywheel leg curl exercise can impose a greater quite homogenous (i.e., all muscle regions) and reproducible (i.e., expected low between-players differences) ST and BFs muscle use in professional football players [16]. By contrast, if the exercise intervention aimed at strengthening and reactivating the commonly injured proximal biceps femoris long head (BFl) during eccentric contractions can benefit for the inclusion of the hip extension conic-pulley exercise [16] or other hip-extension exercise.

## Conclusions

In conclusion, current results indicate an improvement in sprint performance after a football and strength and conditioning training period with or without including the NHE. These improvements are not dependent on the NHEs changes, with no relationships between EHS and sprint performance, and between sprint changes and changes in EHS. Players with extensive experience in NHE could be limited in the NHEs improvements due to the effect of BM on NHEs, affecting the force applied to the dynamometers. Therefore, and in order to continue increasing the EHS in these football players, it will be necessary to employ different hamstring neuromuscular stimulus.

## Supporting information

S1 Dataset Original data. (XLSX)
